# Detecting rare functional variants using a wavelet-based test on quantitative and qualitative traits

**DOI:** 10.1186/1753-6561-5-S9-S70

**Published:** 2011-11-29

**Authors:** Renfang Jiang, Jianping Dong

**Affiliations:** 1 Department of Mathematical Sciences, Michigan Technological University, Fisher Hall, Room 319, 1400 Townsend Drive, Houghton, MI 49931-1295, USA

## Abstract

We conducted a genome-wide association study on the Genetic Analysis Workshop 17 simulated unrelated individuals data using a multilocus score test based on wavelet transformation that we proposed recently. Wavelet transformation is an advanced smoothing technique, whereas the currently popular collapsing methods are the simplest way to smooth multilocus genotypes. The wavelet-based test suppresses noise from the data more effectively, which results in lower type I error rates. We chose a level-dependent threshold for the wavelet-based test to suppress the optimal amount of noise according to the data. We propose several remedies to reduce the inflated type I error rate: using a window of fixed size rather than a gene; using the Bonferroni correction rather than comparing to the maxima of test values for multiple testing corrections; and removing the influence of other factors by using residuals for the association test. A wavelet-based test can detect multiple rare functional variants. Type I error rates can be controlled using the wavelet-based test combined with the mentioned remedies.

## Background

Genome-wide association studies of common mutations in the genome have many important results, but most of the genetic components of the disease risk of common diseases cannot be explained by common mutations. Resequencing studies have identified numerous new rare mutations in the genome. The new hypothesis is that some common diseases may be caused by multiple rare mutations. If each rare mutation has only a small marginal effect, then using methods that are based on single-nucleotide polymorphisms (SNPs) cannot identify them. Therefore we consider a group of SNPs and study the association between the group and the disease. A group can be a gene or a window of consecutive SNPs. Because most common diseases have been studied before, all common mutations with strong marginal effects should have been discovered already. We focus on multiple rare mutations in a gene. Testing multiple SNPs simultaneously for association with diseases can potentially have higher power than testing single SNPs one at a time. The problem here is that a higher number of degrees of freedom is introduced by the increased number of SNPs. Collapsing is a popular idea for dealing with rare variants, whereas a common method is to take a weighted sum [1].

Collapsing methods effectively reduce the number of degrees of freedom. However, useful genetic information may be buried in noise by collapsing all neighboring SNPs together, regardless of whether they are causal or not. Collapsing neighboring genotypes in a gene replaces each genotype at each SNP with the average genotype in a gene, which is the simplest way to smooth multilocus genotypes of an individual. More advanced smoothing techniques include kernel smoothing, Fourier transformation smoothing, and wavelet transformation smoothing. It is interesting to note that the sum of the neighboring genotypes is the first Fourier transformation coefficient of multilocus genotypes. Therefore collapsing methods are a special case of the test based on Fourier transformation [2], in which, instead of genotypes, one analyzes the coefficients of the Fourier transformation of the multilocus genotypes. In collapsing methods, only the first (lowest frequency) Fourier coefficient is analyzed, and all other Fourier coefficients (the high-frequency parts) are ignored. In this sense, the Fourier-based test is a generalization of collapsing methods, whereas the wavelet-based test is an improvement of the Fourier-based test. The wavelet-based tests can be regarded as a generalization of collapsing methods because wavelet transformation is a smoothing technique and collapsing methods are just the simplest way of smoothing.

We earlier proposed a test based on wavelet transformation [3]. The wavelet transformation is designed to handle unsmooth and noisy data. Using this new test has some advantages over the test based on the Fourier transformation because the weighted genotype data are unsmooth and Fourier transformation is mainly for smooth data. By using a process of thresholding, wavelet transformation can effectively reduce the noise, increase the information to noise ratio, and reduce the number of degrees of freedom. This process takes into account the genetic information contained in the linkage disequilibrium structure of the neighboring SNPs. In this aspect, the wavelet-based test is similar to tests based on haplotype similarity [4,5] without the need to estimate unknown haplotypes. For rare alleles and rare haplotypes, such estimations may not always be accurate.

The first step in the wavelet transformation is to transform a noisy, unsmooth weighted multilocus genotype of an individual into its wavelet coefficients. The second step is to threshold the wavelet coefficients by dropping coefficients of small magnitude. Last, the inverse wavelet transformation is used to transform the thresholded coefficients back into a clean and smooth modified weighted multilocus genotype of the individual. Our test is based on the modified weighted multilocus genotype. The choice of threshold procedure is crucial, and we use empirical Bayes thresholding [6]. The threshold procedure is done on an individual basis, so it does not smooth out rare SNPs.

## Methods

In the Genetic Analysis Workshop 17 (GAW17) simulated data there are 697 unrelated individuals consisting of 209 case subjects and 488 control subjects. These individuals were genotyped on 24,487 SNPs in 3,205 genes on 22 chromosomes. There are three quantitative traits (Q1, Q2, and Q4) and a qualitative trait (Affected/Unaffected). Three environmental factors are also given: Sex, Age, and Smoking status. We have requested and obtained the answer document that describes the simulation model.

As many initial tests show, inflated type I error rates are the most serious problem faced by people who are dealing with this data set. The inflated type I errors could be caused by many factors. The first is population stratification. Although population origin was not used in the phenotype simulation, the differences among populations still need to be considered. To tackle this problem, we applied Eigenstrat [7] to remove the effects of population stratification. We used the top *k* eigenvectors obtained by Eigenstrat to adjust genotypes and phenotypes [7]. Let *G* be the genotype matrix, *C* be the matrix of the principal component vectors, *C′* be its transpose, and *Y* be the vector of phenotypes. The adjusted genotype matrix is *G* − *CC′G* and the adjusted phenotype vector is *Y* − *CC′Y*. They are still denoted as *G* and *Y*. We need to determine the number of eigenvectors to be used. The first two eigenvalues (23.138 and 15.087) are much larger than the others, so we let *k* = 2.

After adjusting for population stratification, the type I error rates were still inflated. A relationship between the size of a gene and the type I error rate has been observed before [8]. Most researchers test association between a gene and a phenotype. The advantage of this approach is that higher power is achieved and the results can have biological meanings. However, because the inflated type I error rates are the main concern, we take a different approach. We used eight consecutive SNPs as a window and tested the association between a window of SNPs and a phenotype. As a result, the type I error rate is reduced. Because phenotypes are more likely to be related to nonsynonymous SNPs, it is reasonable to test only nonsynonymous SNPs, which further reduces the type I error rates.

There are three environmental factors: Age, Sex, and Smoking status. Using the first replicate, we test the relationship among environmental factors and phenotypes. Regressing Age on the three quantitative traits yields *p*-values less than 0.0001 (Q1), 0.3437 (Q2), and less than 0.0001 (Q4). So Q1 and Q4 are related to Age, and Q2 is not. Similarly, we found that the relationships between Sex and both Q1 and Q2 are not significant, but the relationship between Sex and Q4 is significant. The relationship between Smoking and Q1 or Q4 is significant, but the relationship between Smoking and Q2 is not. Similar tests between Age, Sex, Smoking, and the qualitative trait show that the Affected status is influenced by Smoking and Age but not by Sex. The results of using logistic regression to test the relationships between Q1, Q2, Q4, and the Affected status illustrate that all three quantitative traits are related to the Affected status. In summary, Q1 is related to Smoking and Age; Q4 is related to Sex, Smoking, and Age; and the qualitative trait Affected is related to Q1, Q2, Q4, Smoking, and Age.

To identify the functional variants for a trait, we need to remove the influences of other factors. Therefore we first adjust each environmental factor for population stratification by *Z* − *CC′ Z*, where *Z* is an environmental factor and *C* is the matrix of the principal component vectors. For quantitative traits Q1, Q2, and Q4 and the qualitative trait Affected, we fit the following linear regression models:

Q1 = *a*_0_ + *a*_1_(Age) + *a*_2_(Smoking), (1)

Q4 = *a*_0_ + *a*_1_(Age) + *a*_2_(Smoking), (2)

Affected = *a*_0_ + *a*_1_(Q1) + *a*_2_(Q2) + *a*_3_(Q4) + *a*_4_(Smoking) + *a*_5_(Age), (3)

where Q1, Q2, Q4, Age, Sex, and Smoking are all adjusted for population stratification. Let Q1, Q2, Q4, and Affected be the residuals of models (1)–(3). We test the association between these residuals and genes (or windows of SNPs). Using residuals of the traits instead of the original traits can further reduce the type I error rates.

When thousands of genes are tested, the problem of multiple testing needs to be addressed. There is a common way to obtain a global *p*-value: One randomly permutes a phenotype *M* times and finds the maximum of a test value on all genes for each set of permuted phenotypes. The global *p*-value of a test value *T* on a gene is the proportion of the *M* maxima that are greater than *T*. To test at a significance level of 0.05, one just needs *M* = 5,000 permutations. This approach works fine when test values on all genes have the same distribution under the null hypothesis. If the distributions are different, taking this approach will reduce power drastically. The type I error rate will be inflated in order to keep a reasonable power (see Table 1). To avoid this disadvantage, we take the following approach. We use a significance level of 0.05. The empirical *p*-values of tests on each window (or gene) are computed by 60,000 permutations. For the Bonferroni correction, the empirical *p*-values are multiplied by the number of windows (or number of genes), which is 1,702 (or 2,342). This multiple testing correction procedure has smaller type I error rates than that obtained using *M* maxima.

**Table 1:** Powers and type I error rates

Window	Q1	Q2	Q4	Affected
16 SNPs				

Power	*FLT1* (0.82) *KDR* (0.17) *ARNT* (0.015) *VEGFC* (0.005)	*BCHE* (0.02) *SIRT1* (0.005) *VNN3* (0.035) *LPL* (0.005) *VLDLR* (0.01) *SREBF1* (0.02)		*HSP90AA1* (0.005) *PRKCA* (0.005)
Type I error	0.085	0.121	0.134	0.099
8 SNPs				
Power	*FLT1* (0.68) *KDR* (0.16)	*BCHE* (0.01) *SIRT1* (0.005)		*HSP90AA1* (0.005) *PRKCA* (0.005)
Type I error	0.035	0.075	0.11	0.05
4 SNPs				
Power	*FLT1* (1.0) *KDR* (0.15)	*RARB* (0.005) *VNN1* (0.025) *VNN3* (0.01) *SREBF1* (0.005)		*SOS2* (0.005)
Type I error	0.247	0.162	0.178	0.150
Genes				
Power	*FLT1* (0.68) *KDR* (0.26)	*GCKR* (0.01) *BCHE* (0.01) *VNN1* (0.02) *LPL* (0.005) *VLDLR* (0.01) *SIRT1* (0.005) *SREBF1* (0.005)		*HSP90AA1* (0.005) *PRKCA* (0.005)
Type I error	0.06	0.21	0.27	0.155
4 less rare SNPs, no adjustments for other factors				
Power	*FLT1* (0.575) *ARNT* (0.015)	*VNN1* (0.005) *VNN3* (0.005) *LPL* (0.015)		
Type I error	0.050	0.136	0.085	0.105

Using a gene as the window does not make any adjustment for population stratification and environmental factors. Values in parentheses are the powers.

To compare these two methods of permutation, we run the following simulation. Consider 100 independent random variables *X*(*i*) for *i* = 1, 2, …, 100. Let *e* be the standard normal random variable. We consider two scenarios. In the first scenario, all *X*(*i*) have the same distributions under the null hypothesis; in the second scenario, the *X*(*i*) have different distributions under the null hypothesis. Under the null hypothesis, in the first scenario, *X*(*i*) = *e*; in the second scenario, *X*(*i*) = *a*(*i*) + *b*(*i*)*e*, where the mean of *X*(*i*), *a*(*i*), is selected from the standard normal distribution, and the standard deviation of *X*(*i*), *b*(*i*), is selected from a uniform distribution on [0.5, 1.5]. Note that in the second scenario the average of *a*(*i*) is 0 and the average of *b*(*i*) is 1. Under the alternative hypothesis, *X*(1) = 3 + *e*, and all other *X*(*i*) are the same as those under the null hypothesis. So *X*(1) is the functional SNP, and all other *X*(*i*) are nonfunctional SNPs.

For each scenario, we take a sample size of 100,000 for each *X*(*i*) under the null hypothesis to mimic results of 100,000 permutations. We then take a sample size of 1,000, *T*(*i*, 1), …, *T*(*i*, 1,000) for each *X*(*i*) under the alternative hypothesis as the test values in 1,000 replicates to compute the power and type I error rates. We compute empirical *p*-values for each *T*(*i*, *s*) using two methods: by comparing to the maxima and by applying the Bonferroni correction. We then use 1,000 replicates to find the power and type I error rates at a significance level of 0.05; the results are given in Table 1. From Table 1, we can see that the second method is robust. As expected, the power of the second method decreases slightly when the distributions of *X*(*i*) are not the same under the null hypothesis. On the other hand, the power of the first method drops to almost 0 when the distributions of *X*(*i*) are not the same under the null hypothesis, and the type I error rate is also inflated. In practice, if we use the first method, the type I error rate has to be inflated substantially in order to increase the power. Table 1 shows the advantage of the second method over the first. The only drawback of the second method is the computational burden. So long as that can be contained, we should use the second method.

The genotypes *AA*, *aA*, and *aa* at each SNP are coded as 0, 1, and 2, respectively, where *a* is the minor allele at the SNP. Because most functional variants are rare alleles, we put a weight on a rare allele. A coded genotype is divided by the square root of *nq*(1 − *q*), where *n* is the total number of individuals and *q* is the estimated minor allele frequency (MAF) of the *i*th SNP [1]. Let *X* be the matrix of weighted genotypes. The *j*th column *X*(*j*) of *X* is the weighted genotype of the *j*th individual. We apply wavelet transformation, thresholding, and inverse wavelet transformation consecutively on vector *X*(*j*), resulting in the modified genotype of the *j*th individual, denoted still as *X*(*j*).

Empirical Bayesian thresholding [6] is determined as follows. Suppose that the sample wavelet coefficients *Z* are from a normal distribution with mean *u*. Suppose that *u* has a prior distribution of (1 − *w*)*d*(*u*) + *we*(*u*), where *d* is an atom probability density function at 0 and *e* is a symmetric density function. Let *u′*(*z*; *w*) be the median of *u* given *Z* = *z*. There exists a function *t*(*w*) such that *u′*(*z*; *w*) = 0 if and only if |*z*| is less than or equal to *t*(*w*). Let the threshold be *t*(*w′*), where *w′* is the marginal maximum-likelihood estimator of *w*.

Consider a window of *m* consecutive nonsynonymous SNPs (or all nonsynonymous SNPs in a gene), which is an *n* × *m* submatrix of *X*, still denoted by *X*. Subtract the mean of the *i*th row of *X* from each entry of *X* such that the mean of each row of *X* is 0. Let *Y* = (*Y*(1), …, *Y*(*n*)) be the traits of *n* individuals adjusted for population stratification. Assume the generalized linear model [9]:(1)

between *X* and *Y*, where *f* is a link function. Let:(2)

be the score statistic for the markers. Let *V*(*j*) be an estimated variance of *U*(*j*) under the null hypothesis. The global score statistic of this window for trait *Y* is:(3)

where *V* is the square root of the sum of all *V*(*j*). We compute *T* for four different traits (Q1, Q2, Q4, and Affected).

## Results

We analyzed 200 replicates. The null hypothesis for a window (or a gene) is rejected if the *p*-value after Bonferroni correction is less than 0.05. This process was done for 1,702 windows (or 2,342 genes) using 200 replicates. For a functional window (which contains functional variants), the power is the number of times the null hypothesis is rejected divided by 200. For a nonfunctional window (which does not contain functional variants), the type I error rate is the number of times the null hypothesis is rejected divided by 200. The power and type I error rates are reported in Table 2.

**Table 2:** Comparison of two permutation methods

Method	Power	Type 1 error
Method 1 (compare with maxima of test values)		
The same distribution under the null hypothesis	0.299	0.049
Different distributions under the null hypothesis	0.005	0.061
Method 2 (Bonferroni correction)		
The same distribution under the null hypothesis	0.283	0.046
Different distributions under the null hypothesis	0.279	0.050

When using eight SNPs as a window and at a significance level of 0.05, the type I error rates for Q1, Q2, Q4, and Affected were 0.035, 0.075, 0.11, and 0.05, respectively. The type I error rates were under control for Q1 and Affected. For the other two traits, the type I error rates were still inflated, although smaller. The wavelet-based test is sensitive to the choice of window size. Using a window size of 4 or 16 led to inflated type I error rates. Instead of consecutive SNPs, if we used all nonsynonymous SNPs in a gene as a window, the type I error rates more than doubled with a small gain in power. This small gain is not worth it. The power on most functional variants was low, although *FLT1* could be consistently identified. Note that the type I error rate for Q1 was 0.035, which means that among 1,691 nonfunctional windows with 200 replicates, a total of 338,200 tests, the null hypothesis was wrongly rejected only seven times.

## Discussion

Using Eigenstrat [7] can remove some effects of population stratification; however, it cannot remove all inflated type I errors. A large number of principal components will not always reduce the type I error rate; sometimes it does just the opposite. The number of principal components used should depend on the distribution of the eigenvalues. In this study, two principal components were much larger than the others and consequently were used. Because the type I error rates seem to increase as the number of SNPs in a gene increases [8], using a window of fixed size instead of a gene as a window can have a lower type I error rate with only a little sacrifice in power. Removing the influences of other factors before identifying the functional variants for a trait can both increase power and reduce the type I error rate. Finally, the choice of the multiple testing correction method will also have an effect on type I error rates. Despite the popularity and convenience of a given test, in general, we cannot assume that the test has the same distribution in all genes (or all windows) under the null hypothesis. The common practice of using the maxima of test values on all 22 chromosomes for multiple testing correction will increase type I error rates. Obtaining *p*-values by permuting phenotypes on each window (or gene) followed by Bonferroni correction is a safer choice.

From Table 2, it seems that the wavelet-based test algorithm has a higher power when testing a gene that contains more functional variants. For example, for the quantitative trait Q1, the genes *FLT1* and *KDR* have 11 and 10 functional variants, respectively, and all other functional genes have fewer than 6 functional variants. For the quantitative trait Q2, the genes *BCHE*, *SIRT1*, and *SRERF1* have 13, 9, and 10 functional variants, respectively; all other genes have fewer functional variants. For the qualitative trait, all genes except one have less than five functional variants; the power of the wavelet-based test on the qualitative trait is lower.

For the GAW17 data set, the wavelet-based test works the best when the window size is 8. All genes except one contain fewer than 14 functional variants. Increasing the window size only increases noise and type I error rates but not the power of the test. When window size is reduced, the test can capture only a part of the functional variants in a gene, which reduces the power. One of the advantages of the wavelet-based test is to take into account the genetic information contained in the linkage disequilibrium structure of the neighboring SNPs. A small window size loses this advantage. With 24,487 SNPs on 22 chromosomes, the data set is sparse. For a denser data set, a larger window size may be needed to capture most of the functional variants in a gene.

When the wavelet-based test is applied to less rare SNPs with MAFs greater than 1% with or without adjusting for population stratification and with or without removing the environmental factors, the power is decreased and the type I error rates are increased in all but one case: a window size of 4 without any adjustments. It seems that for less rare SNPs, a window size of 4 is better than a window size of 8, and not removing environmental factors is better than removing them. Each gene contains only a few functional variants with MAF greater than 1%. The wavelet-based test does not work well under this circumstance.

## Conclusions

As an advanced smoothing technique, wavelet-based tests are a generalization of collapsing methods. A level-dependent threshold can reduce noise and type I error rates. The inflated type I error rate is a serious problem faced by practitioners. This problem has several causes, and one remedy cannot solve it. We suggest the following: using a window of fixed size rather than using a gene as a window; using Bonferroni correction rather than the maxima of test values for the multiple testing correction; removing other influencing factors and using residuals for association tests; and using Eigenstrat [7] to remove some effects of population stratification and choosing the number of principal components according to the distribution of the eigenvalues.

## Competing interests

The authors declare that there is no competing interest.

## Authors’ contributions

RJ participated in the design of the study and performed the statistical analysis. JD conceived of the study and participated in its design and coordination and helped to draft the manuscript. All authors read and approved the final manuscript.
